# Two-Dimensional Wavelike Spinel Lithium Titanate for Fast Lithium Storage

**DOI:** 10.1038/srep09782

**Published:** 2015-05-18

**Authors:** Jiehua Liu, Xiangfeng Wei, Xue-Wei Liu

**Affiliations:** 1 Future Energy Laboratory, School of Materials Science and Engineering, Hefei University of Technology, 193 Tunxi Road, Hefei, Anhui, 230009, China; 2 School of Chemistry and Chemical Engineering, Hefei University of Technology, Tunxi Road No. 193 Tunxi Road, Hefei, Anhui, 230009, China; 3 School of Physical & Mathematical Sciences, Nanyang Technological University, Singapore 637371 Singapore

## Abstract

Safe fast-charging lithium-ion batteries (LIBs) have huge potential market size on demand according to their shortened charging time for high-power devices. Zero-strain spinel Li_4_Ti_5_O_12_ is one of ideal candidates for safe high-power batteries owing to its good cycling performance, low cost and safety. However, the inherent insulating characteristic of LTO seriously limits its high-rate capability. In this work, we successfully synthesize novel wavelike spinel LTO nanosheets using a facile ‘co-hydrolysis’ method, which is superior to molten-salt approach and traditional solvothermal method in some respects. The unique 2D structures have single-crystal framework with shortened path for Li ion transport. As a result, the N-doped 2D wavelike LTO with 0.6 wt.% of ‘carbon joint’ not only exhibits exciting capacity of ~180 and ~150 mA h g^−1^ for fast lithium storage at high discharge/charge rates of 1.7 and 8.5 A g^−1^ (10C and 50C) respectively, but also shows excellent low-temperature performance at −20°C. In addition, the cost may be further decreased due to recycled functional reagents. This novel nanostructured 2D LTO anode material makes it possible to develop safe fast-charging high-power lithium ion batteries.

In principle, the charging-rate capability of safe LIBs depends largely on the performance of anode for lithium storage. High-rate charging produces sudden heating of LIBs because of polarization potential difference of anode, which often brings the fire hazard for high-power batteries with high-cost metallic lithium anode. Graphite-based anode materials for lithium storage are also accompanied by volume swell as well as high-capacity anode materials, such as micro/nano-silicon^1^,^2^, tin dioxide^3^,^4^, cobalt-based oxide^5^, in which case researchers are desired to synthesize safe stable anode materials for high-power LIBs. Compared with the progress of the high-performance cathode materials^6^,^7^,^8^, an urgent task is to develop high-performance anode materials.

Spinel Li_4_Ti_5_O_12_ (LTO) is an ideal host owing not only to its ‘zero-strain insertion’ structural characteristics, but also to its low cost, abundance and environmental benignity^9^. However, the inherent insulating characteristic of LTO seriously limits its high-rate capability, which is one of key parameters to obtain the high-power density of batteries^10^,^11^. Although the addition of conductive additives could improve its surface electronic conductivity for achieving high-rate capability^12^,^13^,^14^,^15^, the cost of LTO materials is increased due to complicated procedures and some expensive additives and templates^16^. On the other hand, LTO materials obtained by molten-salt method often undergo long-time and high-strength milling^17^,^18^. Low surface area of the sintered grains is also a crucial factor to hamper the improvement of rate performance and available capacities for LTO electrode. Therefore, there still remains a challenging issue in developing novel structured LTO materials.

To solve the above problems, nanostructured electrode materials, which possess effective surface area and shortened path for lithium-ion migration, were exploited for increasing the active material/electrolyte interface and shortening the time of Li-ion insertion/extraction. It has been demonstrated that nanostructured LTO materials, such as nanocrystals^10^,^12^,^13^,^19^, nanowires^20^,^21^, hierarchical structure^22^ as well as their composite with conductive additives^12^,^23^,^24^,^25^, could help to fulfill such purpose and also facilitate the electrochemical insertion/extraction of lithium ions. The anode materials with open channels and efficiently exposed facets have a direct influence on the battery’s capacity and recyclability. Ultrathin nanosheets are desired framework for lithium storage owing to large exposed area and short path for Li-ion transport^26^,^27^. However, the nanosheets with smooth facets are easily bonded when overlapping with each other at high temperatures, which leads to a loss of surface area and affect the battery’s performance. Although assembling the nanosheets into the hierarchical structure is an efficient way to increase their surface area, the high porosity of the electrode makes the energy density low^27^,^28^,^29^.

Palm leaves not only own large corrugated surface for photosynthesis but also have stable weather-resistant structure shown in Figure 1a. Inspired by this natural architecture, in this project, we proposed the corrugated 2D nano-architecture (Figure 1b) which may retain big exposed surface area and improve its electrochemical performance. We also focus on the improvement of surface performance with the aid of template and low-cost structure directing agents. It is no doubt a great effort to design and synthesize the wavelike 2D structure in a facile system. We herein report a facile ‘co-hydrolysis’ method to synthesize wavelike LTO nanosheets on a large scale for the first time. This novel method can avoid high-temperature processes and long-time milling for molten-salt method^17^ and exhibit higher yield than traditional solvothermal methods. As a result, the 2D wavelike LTO with 0.6 wt.% ‘carbon joint’ exhibits excellent capacities of ~180 and ~150 mA h g^−1^ at discharge/charge rates of 10C and 50C respectively.

## Results and discussion

**Figure 2:**
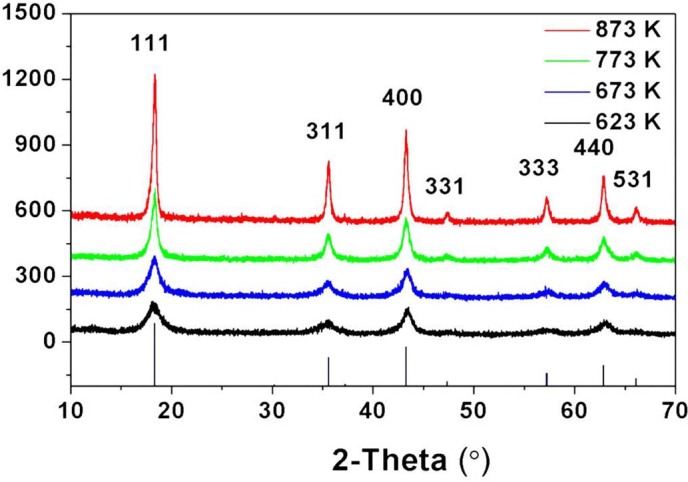
XRD patterns of the LTO samples annealed at 350, 400, 500, 600°C respectively.

Amorphous lithium titanate could be firstly obtained at lower temperature due to the uniform distribution of lithium and titanium formed by rapid hydrolysis. XRD was employed to examine crystallization process with different temperatures. XRD patterns were obtained for the LTO samples annealed at 350, 400, 500 and 600°C, as shown in Figure 2. All Bragg peaks of the obtained samples are consistent with those of spinel Li_4_Ti_5_O_12_ phase (space group Fd3m (227), JCPDS no. 049-0207). The average crystal size of the sample annealed at 350°C is only ~5 nm based on full width at half maximum of peak (111). The crystal sizes rise to ~15 and ~41 nm while increasing temperature to 500 and 600°C respectively.

**Figure 3:**
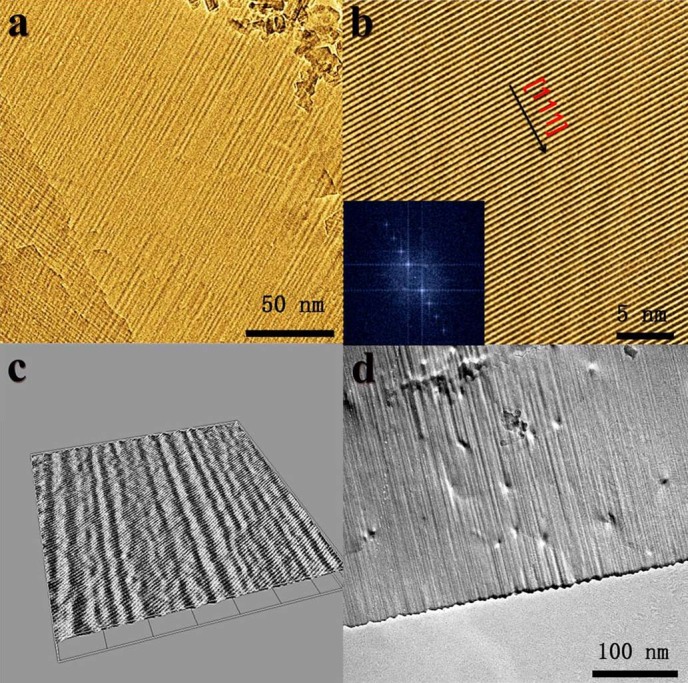
a) TEM image of wavelike LTO nanosheet annealed at 500°C; b) HRTEM image and its fast Fourier transform pattern (inset) obtained from (a); c) 3D image of (b); d) TEM image of wavelike LTO nanosheet annealed at 600°C.

The morphology can be observed directly with the help of TEM images. Figure S1 clearly shows that LTO sample annealed at 500°C is 2D structure with a rough surface. The wavelike LTO nanosheets have a scale about 10 nm in thickness and 400–1000 nm in width/length. Some smaller nanocrystals take the role of pillars to partly avoid overlap to some extent at high temperature. The wavy framework with non-periodic corrugation is also directly observed in Figure 3a and 3b. In the high-magnification TEM image (Figure 3b), it can be observed that one set of big-area lattices are present. The lattice on the exposed facet displays an equal interfringe spacing of 0.48 nm along the [111] axis, which offers the enough space for zero-strain insertion of lithium ions with a diameter of 0.12 nm. Its fast Fourier transform pattern (inset of Figure 3b) indicates single-crystal framework corresponding to LTO planes zone. The wavelike framework is also supported by 3D image (Figure 3c) of LTO nanosheet (Figure 3b) obtained by Image J software analysis. Although its crystal size becomes bigger supported by XRD analysis, the wavelike framework is still maintained when annealing at 600°C as shown in Figure 3d.

**Figure 4:**
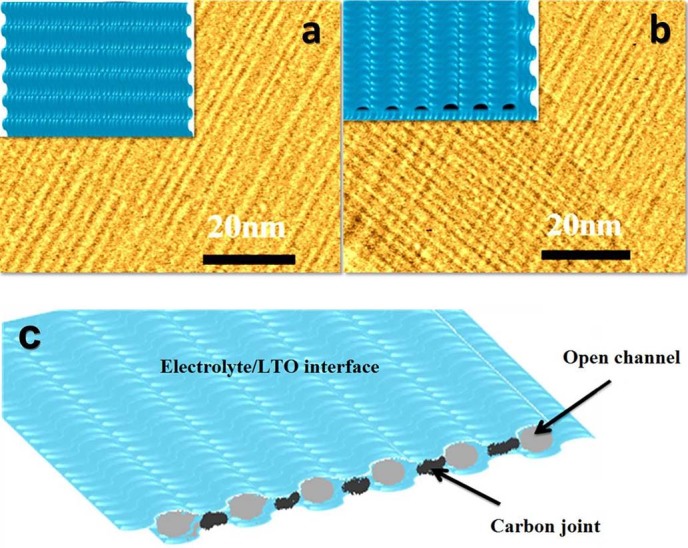
TEM images and scheme of single-layer (a) and overlapped LTO nanosheets (b); c) Schematic formation of ‘carbon joint’ between the overlapped wavelike LTO nanosheets.

Surface properties of LTO were also detected via N_2_ adsorption–desorption method. Its N_2_ adsorption–desorption isotherms and pore size distribution were measured for LTO sample (Figure S2). The surface area and total pore volume of LTO nanosheets annealed at 500°C are 206 m^2^ g^−1^ and 0.166 cm^3^ g^−1^ respectively, which were obtained from N_2_ adsorption-desorption isotherm at 77 K. To our best knowledge, this surface area is the largest among all reported results of LTO nanosheets in Table S1^29^,^30^,^31^,^32^. From the pore size distribution curve of LTO, three peaks are present at 1.1, 2.9, 4.6 nm respectively, which are corresponding to its TEM image with different corrugation spacing. Even annealing at 600°C in absence of oxygen, the sample still has a large surface area of 172 m^2^ g^−1^. The large area also supported TEM analysis for the surface on overlapped nanosheets and their schemes as shown in Figure 4a and 4b. The overlapped nanosheets can still retain their exposed surface owing to the unique wavelike surface structure, which can be directly observed.

Microenvironments of LTO samples were further explored by solid-state NMR.^7^Li magic angle spinning (MAS) spectra of LTO samples annealed at 200 and 500°C were recorded on a JNM-ECA400 spectrometer at 100.5 MHz and chemical shifts were referenced to a 1.0 M lithium chloride aqueous solution. As shown in Figure S3, the resonance of LTO annealed at 200°C is actually composed of two resonances, a broad resonance due to Li on 16d and a narrow resonance due to Li on 8a^33^,^34^. With increasing the temperature, Li on 16d jumped on 8a and the broad resonance (Li on 16d) disappears. Meanwhile, the spinel LTO could be obtained from amorphous lithium titanate. The cubic local environment of the 8a is consistent with the XRD analysis of cubic spinel LTO. The reversible capacity of electrode material is limited by the amounts of protons attached to the surface or the bulk, which is irreversibly replaced by lithium ions when discharging the battery.^35^ In fact, different amount of protons were present in the LTO nanosheet samples as proved by CP/MAS ^1^H NMR spectra of LTO samples (Figure S4). With increasing the temperature, the amount of proton residue decreased. It gives a possible way to improve the reversible capacity of LTO samples. Following our further study we focus on the LTO samples annealed at 600°C due to improving the reversible capacity and reducing the influence of flatulence.

The actual formation mechanism of the wavelike LTO nanosheets is not yet clarified. The possible one is proposed and the schematically illustrated in Scheme S1. First, amorphous lithium titanate can be fast formed because of the co-hydrolysis of lithium and titanium resources. And what is more, low-cost N,N-dimethylethanolamine (DMEA) acts as the effective, shortest bifunctional structure directing agent with the ammonium group (big head) and hydroxyl group (small tail). The two kinds of functional groups could easily form strong intermolecular hydrogen bonds with Ti–OH groups, which may form a curve by well-organized organic molecular structure and lead to the formation of wavelike structure. Then wavelike amorphous titanate nanosheets were formed along 2D plane direction. After removal of solvent, single-molecule layer of DMEA is still firmly held on the surface of amorphous titanate nanosheets below 250°C and is decomposed at 300°C or above supported by thermogravimetry (Figure S5). Therefore, after crystallized in air, white powder is obtained due to the removal of the organic layer. However, the obtained sample is a gray powder after annealed in the absence of air. We hypothesized that ‘carbon joint’ was formed owing to the carbonization of single-molecule DMEA layer. However, the ‘carbon joint’, which stemmed from the carbonization of single-molecule DMEA layer, is so low and thin that it is hardly detected by HRTEM image. The 0.6 wt.% and 0.1 wt.% of exact carbon and nitrogen contents are finally detected by trace elemental analysis. To facilitate the understanding, we provided the schematic formation of ‘carbon joint’ between the overlapped wavelike LTO nanosheets in the absence of air in Figure 4c.

**Figure 5:**
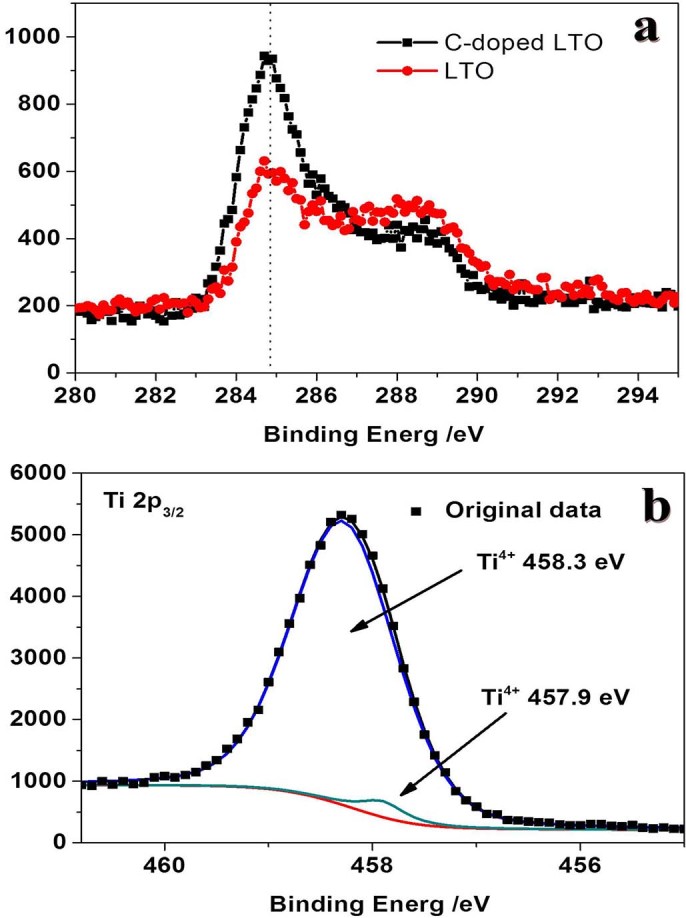
C1s (a) and Ti2p (b) XPS spectra of Carbon coated LTO sample. The samples were calibrated by adventitious carbon at 384.8 eV.

To further confirm the microenvironment of carbon and titanium, the surface chemical compositions of LTO samples were determined by X-ray photo-electron spectrometer (XPS). The carbon content of CLTO with doped nirtogen has a significant increase than adventitious carbon at 384.8 eV (Figure 5a) as an auxiliary evidence for carbon analysis. The exact contents of carbon cannot be obtained, because the intensity depends not only on the amount of carbon in the sample but also on the amount of loading sample. Moreover, in Figure 5b, two kinds of Ti^4+^ peaks on CLTO at 457.8 and 458.3 eV are due to the different microenvironment between big exposed surface and inner crystal, which may improve the surface performance for lithium storage. The peak at 457.8 eV may be attributed to Ti–N bonding, implying that the conductivity could be improved on the surface. The direct evidence is N mapping of CLTO naosheets provided in Figure S6. No Ti^3+^ was detected in CLTO sample^36^.

**Figure 6:**
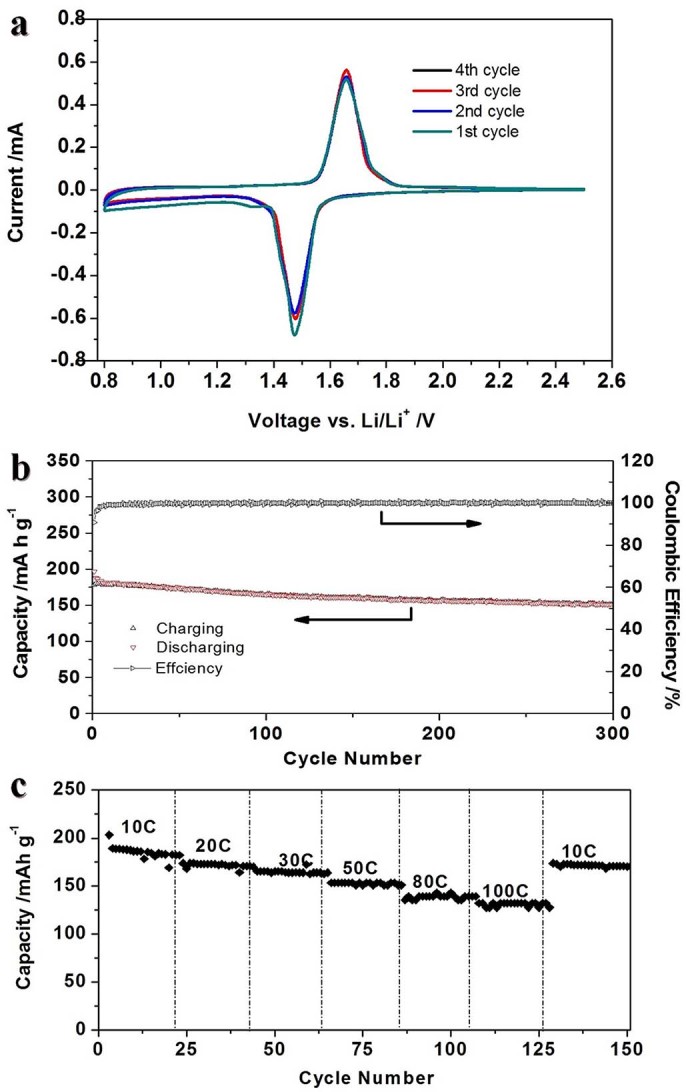
a) Cyclic voltammogram of CLTO at a scan rate of 0.2 mV s^−1^; b) Cycling performance of the CLTO at a constant current drain of 10C and the corresponding Coulombic efficiency; c) Cycling performance at different charge/discharge rates of 10C–100C.

The electrochemical studies of the CLTO and LTO annealed at 600°C were obtained using coin cells with metallic Li slices serving as both the counter and reference electrodes at room temperature. The cyclic voltammetric curve (Figure 6a) of electrode made of CLTO was determined at a scanning rate of 0.2 mV s^−1^. The typical current peaks of LTO are obvious at voltages of ~1.47 V and ~1.66 V. Its insertion-deinsertion peak separation of 0.19 V is smaller than 0.24 V of LTO (Figure S7) at the same scan rate, which reflects that CLTO may have low polarization.

The cycling performance and needed time of CLTO was studied as a high-rate anode material. Figure 6b shows the cycling performance of the CLTO at a current drain of 10C. High capacities of above 180 mA h g^−1^ were obtained in first 10 cycles. After 300 charge/discharge cycles, a reversible discharge capacity as high as 151 mA h g^−1^ can still be retained and its Coulombic efficiency almost approaches 100%. The improvement of capacity at high rate is due to large electrolyte/electrode interface (grain boundaries) that leads to pseudocapacitive insertion/extraction^37^. The rate performance of the CLTO at 10C – 100C was further investigated as shown in Figure 6c and clearly demonstrates excellent cycling performance at all current rates. At 20C and 30C, the discharge capacities are around 171 mA h g^−1^ and 164 mA h g^−1^ respectively, which is very close to its LTO theoretical capacity^38^. It can still retain 151 mA h g^−1^ at 50C. Even at the highest rate of 100C (17 A g^−1^), a capacity of 122 mA h g^−1^ can be held. Evidently, the electrochemical performance of CLTO is one of best results in all reported LTO and TiO_2_ nanomaterials. The discharging/charging time of CLTO nanosheets shows that this material is an ideal anode for ultrafast charging LIBs. The full charging time at 10C, 20C, 30C are shortened to ~6.5, 3, 2 minutes respectively. At higher rates of 80C–100C, the charging time needed is only one minute or less.

As a comparison task, the electrochemical performance of LTO was also studied at high rates from 10C to 100C (Figure S8). It exhibits a high capacity of 165, 147, 129, 101 and 75 mA h g^−1^ at high discharge/charge rates of 10C, 20C, 30C, 50C, 80C respectively, which is also superior to the most recently reported LTO materials^13^,^39^,^40^,^41^,^42^. Therefore, the unique surface properties of LTO are conducive to increasing high-rate performance. Compared with LTO nanosheets, the improvement of high-rate performance of CLTO should owe to the ‘carbon joint’ formed at adjacent interface of LTO, by which the surface insulating characteristic of LTO may be improved with the assistance of ‘carbon-joint’ and doped nitrogen.

**Figure 7:**
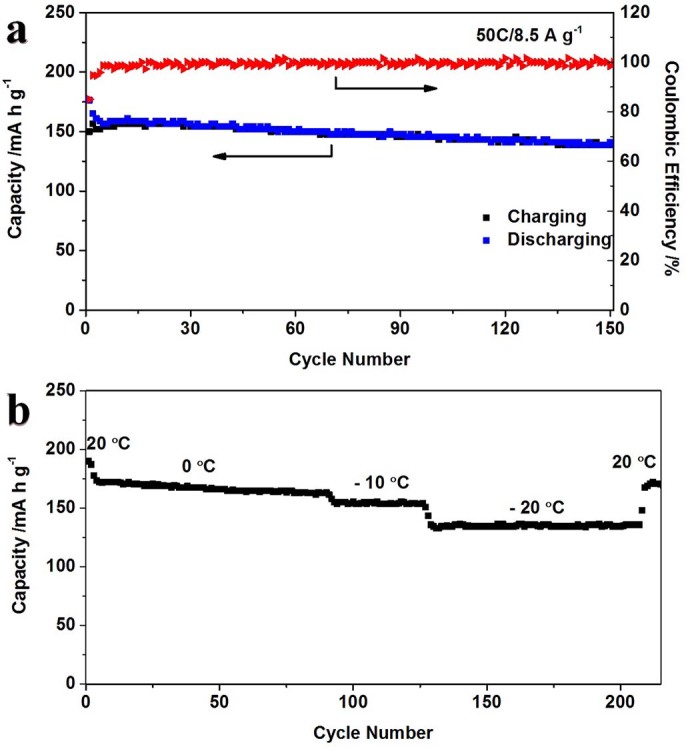
a) Cycling performance of the CLTO at a constant current drain of 50C and the corresponding Coulombic efficiency; b) Cycling performance at a constant current drain of 10C at different temperatures from −20 to 20°C.

Moreover, charge/discharge experiment at 50C was conducted to investigate its high-rate capacities and cycling performance (Figure 7a). After 150 full charge/discharge cycles, their discharge capacity of 140 mA h g^−1^ is remained and its Coulombic efficiency is also close to 100%. The electrochemical studies demonstrated that the wavelike CLTO nanosheets exhibit an excellent reversible capacity, stable cycling performance, and superior high-rate capability. Furthermore, low-temperature and high-rate performance of CLTO was investigated at different temperatures between –20 and 20°C. Figure 7b exhibits excellent capacities of 172, 154 and 135 mA h g^−1^ at 0, −10 and −20°C respectively when charging/discharging at a constant current drain of 10C, its capacity can return to 170 mA h g^−1^ in spite of more than 210 cycles while temperature rising to 20°C.

## Conclusions

On the basis of the above analysis, the wavelike CLTO nanosheets with doped nitrogen can provide a short path for lithium-ion migration and a big electrolyte/electrode interface for lithium insertion. This kind of LTO materials exhibits excellent capacities, good cycling performance and superior low-temperature performance at high-rate work. We forecast the wavelike LTO materials with ‘carbon joint’ have extensive application prospect for ultrafast charging LIBs and hybrid super-capacitors. We believe that the in situ ‘co-hydrolysis’ method is an alternative approach to develop new high-performance electrode materials.

### Experimental Section

#### Preparation of samples

Wavelike LTO nanosheets were synthesized by a facile hydrothermal and co-hydrolysis method (Equation 1 and 2). In a typical route, 0.04 mol of metallic lithium was put into 20 g of DMEA in an ice-bath to form a clean solution. The byproduct (H_2_) may be used as fuel to heat the following hydrothermal reaction. 17 g (~0.05 mol) of tetrabutyl titanate was then added to form single-phase solution with a Li/Ti ratio of 4:5. After that, the obtained solution was added into Teflon-lined stainless steel reactor and H_2_O (6–15 ml) was added, before heated at 160°C for 24 hours. After reaction, the white solid samples were obtained by directly filtrating, drying at 150°C and then annealing at 350–700°C for 2 hours. The LTO nanosheets with carbon joint were obtained with same conditions, except annealing at 600°C in the absence of oxygen. Furthermore, the reagents including DMEA and 1-butanol could be easily removed and reused for next cycle.
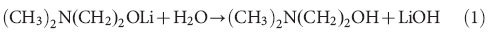

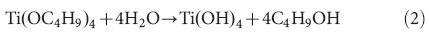


#### Characterizations

The X-ray diffraction (XRD) patterns were performed with a D8 diffractometer with Cu-Kα radiation (λ = 1.54056 Å). TEM were obtained with JEOL JEM-1400 and JEOL 2100F. N_2_ adsorption-desorption isotherms were conducted at 77 K on a Micromeritics Tristar 3000 analyzer. The BET surface areas and pore-size distribution curves were concluded using adsorption data. ^7^Li, and ^1^H Cross Polarization/Magic-Angle Spinning (CP/MAS) or MAS NMR measurements, a JNM-ECA400 spectrometer was used at 100.5 and 400.0 MHz respectively. X-ray photoelectron spectroscopic (XPS, KRATOS, AXIS ULTRA DLD) measurements were carried out by using a monochromated Al Kα (1486.7 eV) X-ray source at power of 150 W (15 kV × 10 mA). The XPS analysis was carried out at room temperature under a typical pressure in the range of 1.0 e^−9^−5.0 e^−9^ Torr at take-off angle relative to the surface holder of about 90 °. Thermogravimetric analysis was determined using a thermal gravity analyzer (TGA) at a temperature-rise rate of 10 K min^−1^ from room temperature to 800°C under a continuous air flow. Carbon trance analysis is determined using EuroVector Euro EA elemental analyzer.

#### Electrochemical tests

The electrochemical tests were performed using coin cells with lithium serving as both the counter and reference electrodes under room temperature. The working electrode was composed of 70–80 wt.% of the active material, 10–20 wt.% of conductivity agent (carbon black, Super-P-Li), and 10 wt.% of binder (polyvinylidene difluoride, PVDF, Aldrich). The electrolyte used was 1 M LiPF6 in a 1:1 (w/w) mixture of ethylene carbonate and diethyl carbonate. Cell assembly was carried out in an Argon-filled glove box. Cyclic voltammetry (CV, 0.8–2.5 V, 0.2 mV s^−1^) was performed using an electrochemical workstation (CHI 760D). Galvanostatic charge/discharge cycling was conducted using a battery tester (NEWAER) at different current rates of 10C–100C, where 1C = 170 mA g^−1^.

## Author Contributions

J.H. and X.-W. designed this work; Experiments were performed by J.H. and X.F. and data were analysed by all authors. J.H. and X.-W. wrote the paper. All authors reviewed the manuscript.

## Additional Information

**How to cite this article**:Liu, J., Wei, X., &Liu, X.-W.Two-Dimensional Wavelike Spinel Lithium Titanate for Fast LithiumStorage. *Sci. Rep.* **5**, 9782; doi: 10.1038/srep09782 (2015).

## Supplementary Material

Supplementary Information SUPPLEMENTARY INFO

## Figures and Tables

**Figure 1:**
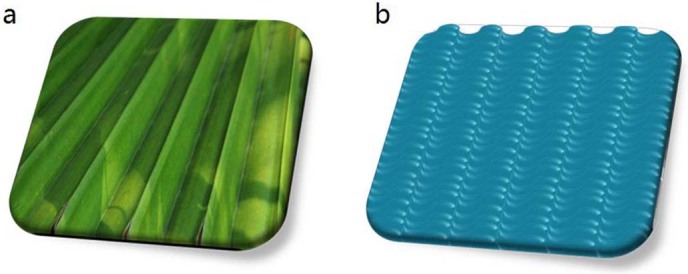
Structures of natural palm leaf (a) and proposed wavelike nanosheet (b).
