# Supplementary material for: Two-Dimensional Wavelike Spinel Lithium Titanate for Fast Lithium Storage
Source: Sci Rep. 2015 May 18;5:9782. doi: 10.1038/srep09782 (PMC4434912; doi:10.1038/srep09782)
Supplement: Supplementary Information — SUPPLEMENTARY INFO [file srep09782-s1.pdf]

Supplementary Information for

Two-Dimensional Wavelike Spinel Lithium Titanate for Fast Lithium Storage

Jiehua Liu,*[a] Xiangfeng Wei,[ab] and Xue-Wei Liu*[c]

[a] *Future Energy Laboratory, School of Materials Science and Engineering, Hefei University of Technology, 193 Tunxi Road, Hefei, Anhui, 230009, China E-mail: liujh@hfut.edu.cn*

[b] *School of Chemistry and Chemical Engineering, Hefei University of Technology, Tunxi Road No. 193 Tunxi Road, Hefei, Anhui, 230009, China*

[c] *School of Physical & Mathematical Sciences, Nanyang Technological University, Singapore 637371 Singapore E-mail: Xuewei@ntu.edu.sg*

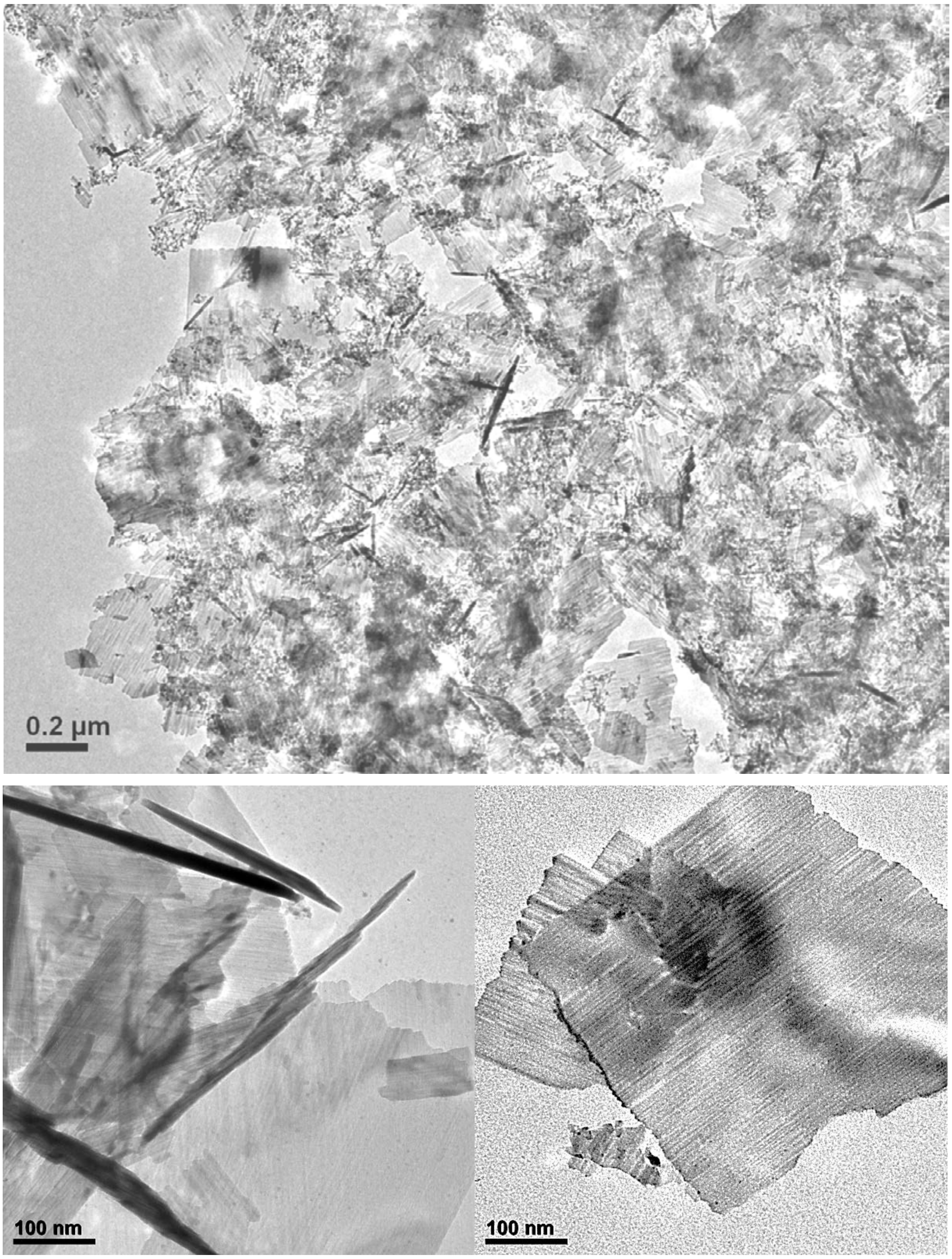

Figure S1. TEM images of wavelike LTO nanosheets annealed at 600 ºC.

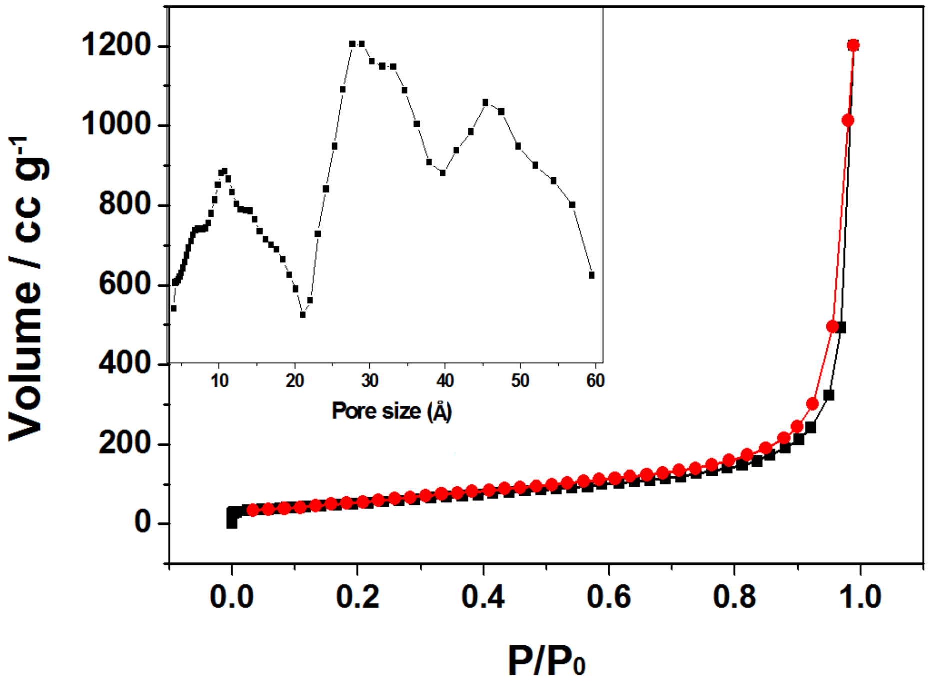

Figure S2. N_2 adsorption-desorption isotherms of LTO sample annealed at 500 ºC and its pore size distribution (inset). 1 cc = 1 cm^{-3}.

Table S1: The surface area of different structures based on LTO nanosheets

No	Materials	Surface area	References
**1**	Wave-like LTO nanosheets	206 m^2 g^{-1} at 500 ºC<br>172 m^2 g^{-1} at 600 ºC	This work
**2**	$Li_4Ti_5O_{12}$ hollow microspheres	131 m^2 g^{-1} at 500 ºC	Electrochim. Acta 54, 6244 (2009)
**3**	$Li_4Ti_5O_{12}$ microspheres	107 m^2 g^{-1}at 700 ºC	*Electrochim Acta* **151**, 502 (2015).
**4**	$Li_4Ti_5O_{12}$ nanosheets	15.5 m^2 g^{-1} at 700 ºC	J. Mater. Chem. A 1, 14618 (2013).
**5**	$Li_4Ti_5O_{12}$ nanosheets	139 m^2 g^{-1} at 550 ºC	Electrochim. Acta 55, 6596 (2010).

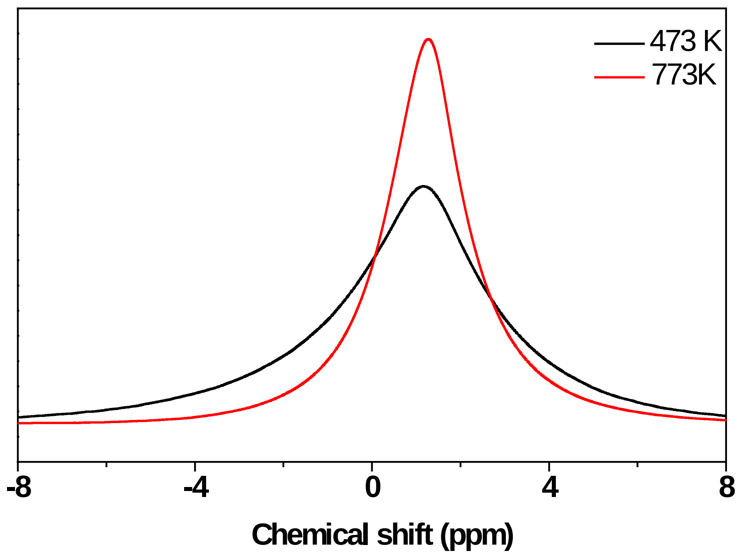

Figure S3. Magic angle spinning solid-state ^{7}Li NMR spectra of LTO samples annealed at 200 and 500 ºC.

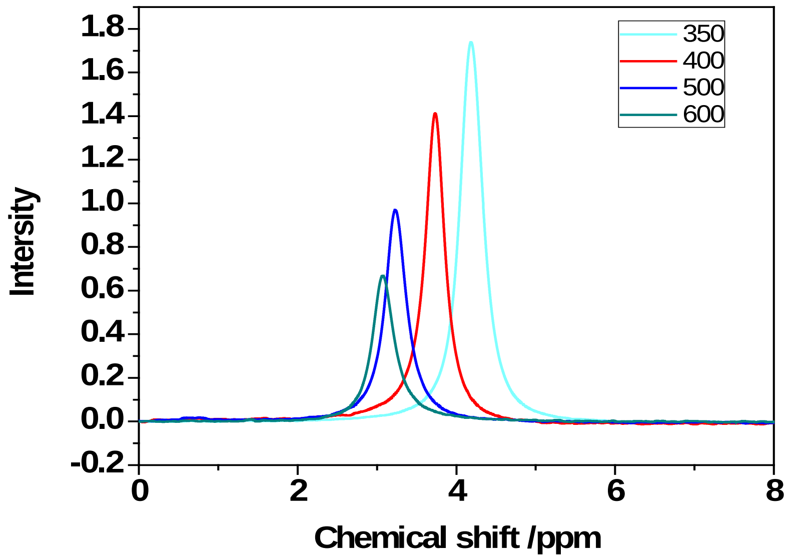

Figure S4. Cross polarization /magic angle spinning solid-state ^{1}H NMR spectra of LTO samples.

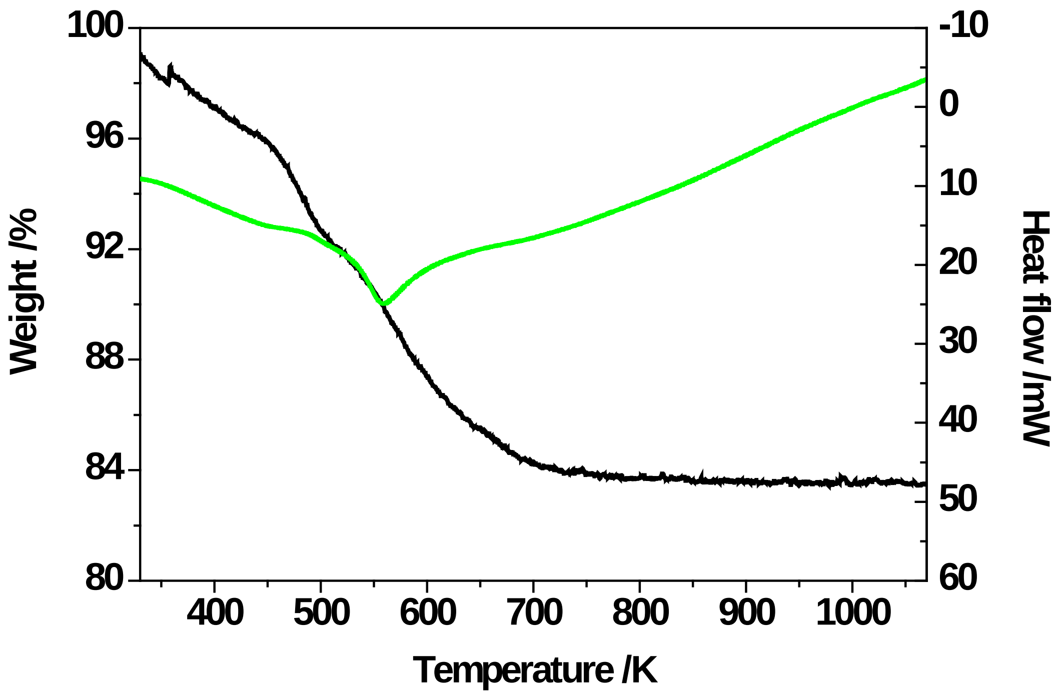

Figure S5. TGA curves of as-synthesized sample at a temperature-rise rate of 10 ºC min^{-1}.

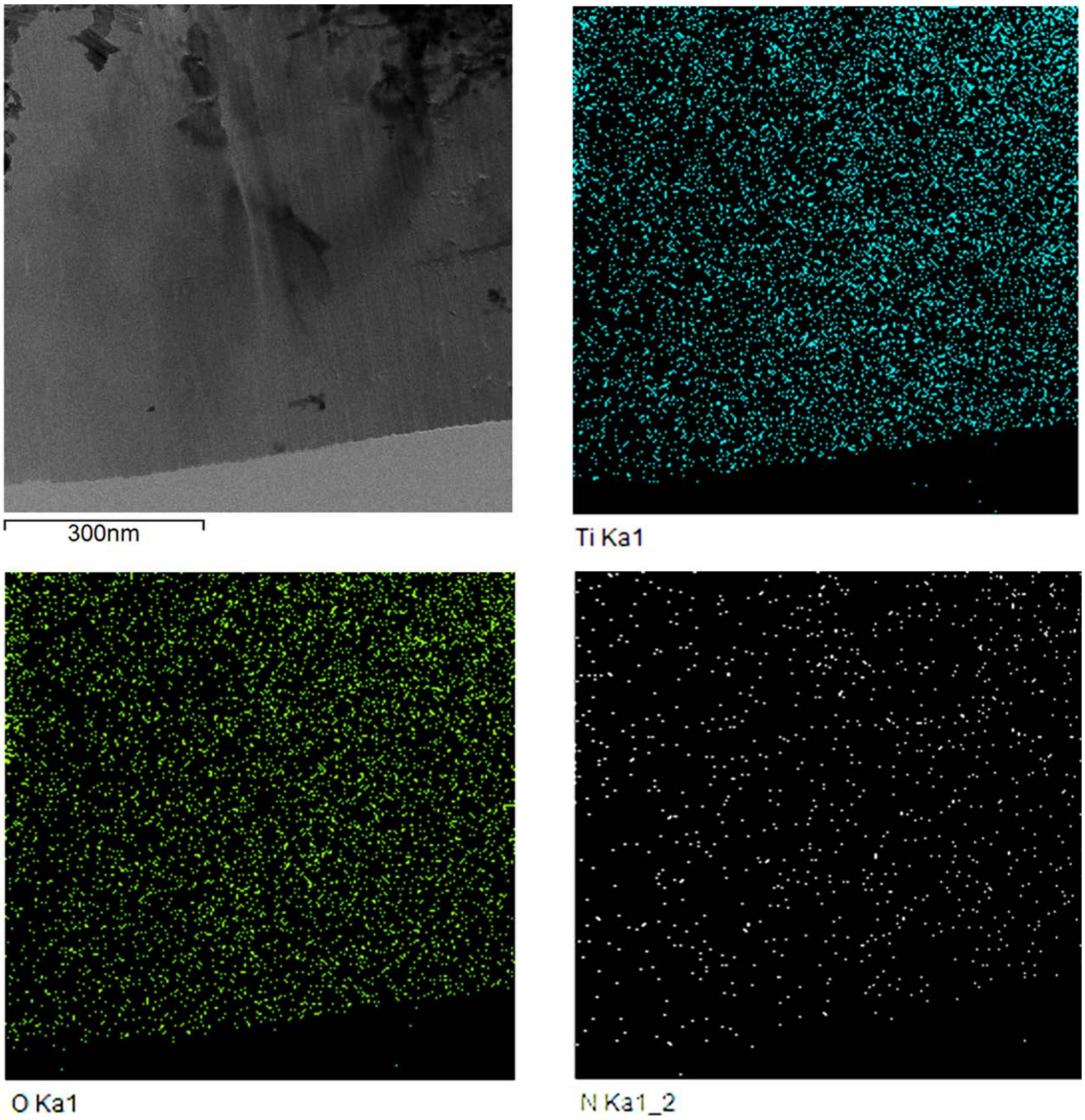

Figure S6. STEM image of CLTO nanosheet and its element mappings of Ti, O and N respectively.

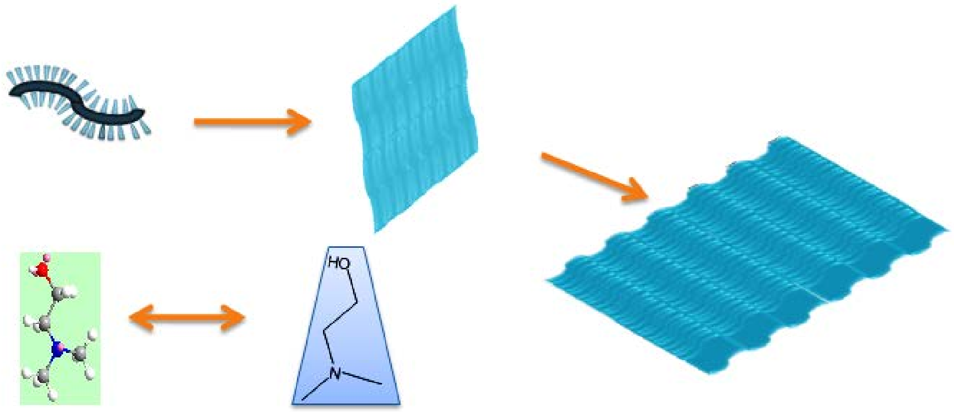

Scheme S1. Formation mechanism of wavelike LTO nanosheets

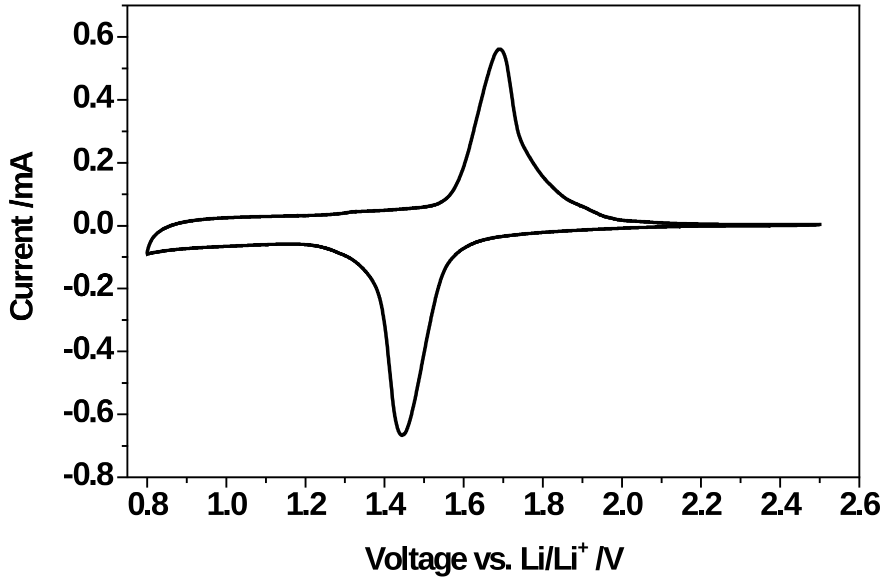

Figure S7. Cyclic voltammogram of LTO nanosheets at a scan rate of 0.2 mV s^{-1}.

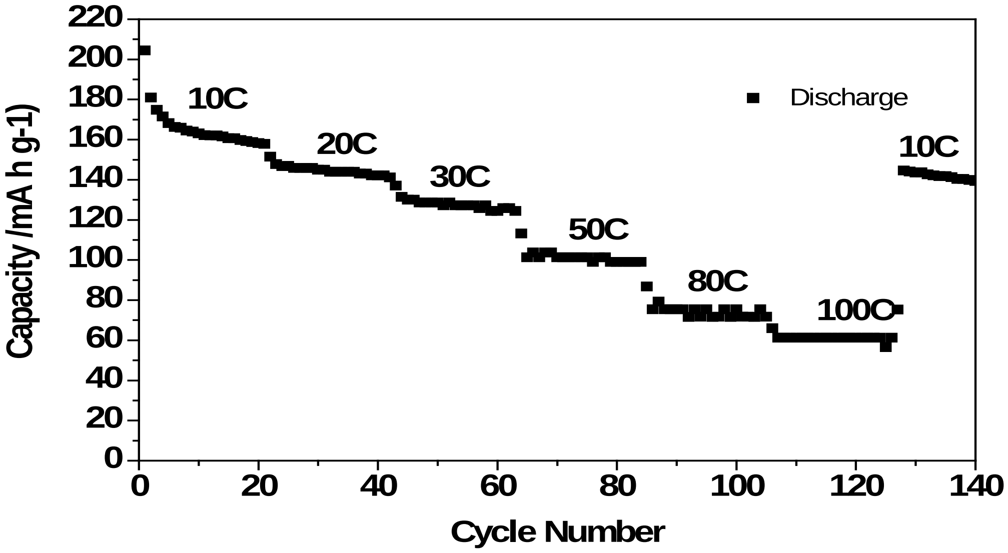

Figure S8. Cycling performance of LTO nanosheets at different charge/discharge rates (10C-100C).
